# Assessment of the price-efficacy relationship for multiple brands of ceftriaxone sodium in Kabul: a cross-sectional study

**DOI:** 10.1186/s13104-016-1904-y

**Published:** 2016-02-12

**Authors:** H. M. Naimi, H. Rasekh, M. Haem Rahimi, H. Yousofi

**Affiliations:** Department of Microbiology, Faculty of Pharmacy, Kabul University, Jamal Meena Street, Kabul, Afghanistan

**Keywords:** Ceftriaxone sodium, Antibiotics, Afghanistan, Medicine, Pharmacy, Cost, Price, Efficacy, *Staphylococcus aureus*, Minimal bactericidal concentration (MBC), Clinical practice

## Abstract

**Background:**

Most medicines are imported for health service practices in Afghanistan. A major concern for patients and practitioners in Kabul is the wide brand assortment and price range choices for the same drug. Ceftriaxone sodium is a broadly used antibiotic for infections caused by certain types of gram-positive and gram-negative bacteria. It is available in Kabul in a range of brands and prices. The objective of this study was to assess the relationship between cost/brand name and efficacy of this antibiotic.

**Methods:**

40 brands of ceftriaxone, obtained from Kabul’s main pharmacy, were derived from 12 countries including Pakistan, Turkey, India, and China. Ten samples/brand were tested for efficacy by the minimal bactericidal concentration assay against a sensitive strain of *Staphylococcus aureus* according to the Clinical Institute and Laboratory Standards Protocols. Efficacy data were obtained by inoculating suspensions of *S. aureus* grown in Mueller–Hinton medium with various concentrations (6.25–800 mcg/ml) of each brand followed by incubation at 37 °C for 18–24 h. Aliquots of inoculated cultures were transferred to agar plates, incubated at 37 °C for 18–24 h and visible colonies counted. Results were analyzed using ANOVA, Student’s t test, and Pearson correlation by SPSS 19. A *p* value ≤ 0.05 was considered statistically significant.

**Results:**

Ceftriaxone sodium price varied from 20–270 Afghanis/brand (average price = 69.80 Afghanis/brand). Of the 40 brands tested, 10 (25 %) were not registered with the General Directorate of Pharmaceutical Affairs of the Ministry of Public Health in Afghanistan. More importantly, we observed no statistically significant difference in efficacy against *S. aureus* among these brands (*p* = 0.59).

**Conclusions:**

Our study showed no significant correlation among price, brand, and efficacy of ceftriaxone sodium against *S. aureus*, an important consideration when treating *S. aureus* infection in Afghanistan and elsewhere. Differences in brand prices are likely due to other factors including manufacturing and exportation costs, regulations of good manufacturing practice and seller’s profit ceiling and patient preferences. Based on our results, we suggest that further chemical and clinical studies of ceftriaxone sodium brands are warranted and recommend that physicians consider alternative cost-effective generic brands in patient prescriptions.

## Background

Efficacy, availability and cost of medicines are a serious concern in health care systems globally. Substandard and counterfeit clinically used drugs that compromise the health of patients continue to overwhelm markets world-wide and is especially a problem in developing countries [1]. Lack of rigorous quality assurance measures in many developing countries including Afghanistan exacerbate the problem and lead to increased incidences of many diseases with devastating consequences on health care infrastructures.

Quality, efficacy and safety must be primary concerns for health care systems world-wide [2]. Yet, contaminating toxic chemical substances, inadequate and inconsistent drug supply mechanisms, and lower-active ingredients doses, are all factors associated with counterfeit/substandard treatments that diminish quality [3] and are risk factors for anti-microbial resistance, organ failure, and increased mortality rates globally [4, 5].

Afghanistan depends on imports of nearly all medicines since its pharmaceutical industries lack the capacity to adequately meet the population’s health care needs. According to the World Health Organization [6], the Ministry of Public Health of Afghanistan is not equipped to effectively control the quality of medical drugs entering the market as regular and periodic quality-control mechanisms necessary to do so are lacking. For example, well-equipped laboratories and well-trained human resources are essential for efficient registration and licensing of imports. Afghanistan’s national medicines quality control laboratory operates under challenging conditions in dilapidated structures, which are not internationally accredited. The UK Department for International Development states that the amount of both donated and imported medicines by private companies has increased dramatically since 2002 in Afghanistan. Furthermore, the amount of illegally imported medicines has risen in the private sector, which further compounds the problem in an already substandard health care situation [7].

Most private importers in Afghanistan who procure medical drugs are located in Kabul city. Considerable difference in prices of various brands of the same drug in the city has raised the question in the scientific community of whether price equates quality. According to the Afghanistan National Medicine Law any registered internal or external company can import registered medicines without considering price differential ranges among brands for the same item [6]. Given the large number of clinically used drugs and the increasingly heavy trafficking of these substances, it is a daunting task for the Ministry of Public Health to identify imports and control efficacy, safety, quality, price and stem the flow of counterfeit medicines in Afghanistan.

One of the main classes of antibiotics used widely in clinics internationally are the third-generation cephalosporins which are effective against certain gram-positive and gram negative bacteria. These antibiotics are used to treat a wide range of infectious diseases including pneumonia, urinary tract infections, meningitis, peritonitis, biliary infections, surgical prophylaxis, prophylaxis of meningitis and gonococcal infections [8]. Ceftriaxone sodium, a member of the third generation cephalosporins, is a drug of choice in the clinical setting to treat such infections because of increased potency and longer half-life [8].

Thus far, comprehensive studies have not been carried out in Afghanistan to assess the relationship between efficacy and price among the many brands of ceftriaxone sold in Kabul pharmacies. The main objective of this study was to determine whether comparative in vitro efficacy contributed to variation in prices in forty different brands of ceftriaxone available in Kabul to promote rational use of cost-effective brands of the drugs.

## Methods

This study was conducted in the microbiology laboratory of the faculty of pharmacy of Kabul University within a period of 6 months. A selection of forty different brands of ceftriaxone sodium was purchased as 1 g/vial/sample from the Kabul private medicine market (Kabul pharmacy), the main wholesaler market of medicines in Kabul city and a distributor of medical drugs throughout the country. This drug is only available as an injectable. Ten samples of each brand of ceftriaxone sodium was tested to compare efficacy with samples of others brands in the group. Each brand was numerically coded to obscure name and price to maintain a blind study. All brands were purchased at the community wholesaler’s fixed rates/drug. All drugs were stored at 25–30 °C until experimental analyses. Drugs that had reached the expiration date were not used in this study.

A susceptible strain of *Staphylococcus aureus* (*S. aureus*; American Type Culture Collection #29213) was used to determine comparative in vitro anti-bacterial activity of each sample/drug/brand using the minimal bactericidal concentration (MBC) of each brand toward *S. aureus* according to the Protocols of the Clinical Laboratory Standards Institute [9]. This *S. aureus* (ATCC 292) strain was used in the study because it showed susceptibility to ceftriaxone and its genotype was confirmed as wild-type by the French Medical Institute in Kabul. In addition, ceftriaxone has been effectively used to treat various clinical conditions caused by *S. aureus*.

All culture media were prepared according to the manufacturer’s (Difco) instructions and all *S. aureus* cultures were maintained in identical growth conditions.

Briefly, we cultured −20 °C frozen *S. aureus* stock on Tryptose blood agar plates (Difco) at 37 °C for 18–24 h. After this time, 1–2 separate colonies of *S. aureus* were transferred into 5 ml normal saline to make a turbidity of 1 McFarland bacteria suspension. We then used 10 µl of this suspension to inoculate test tubes containing 2 ml Muller Hinton broth (Difco) mixed with a ceftriaxon sample with doses ranging from 6.25 to 800 mcg/ml drug sample/brand and cultures subsequently incubated at 37 °C for 18–24 h. Positive Control samples were not treated with ceftriaxone while negative control samples did not contain bacterial suspension. 10 μl of drug-inoculated *S. aureus* cultures without visible growth were next streaked onto Muller Hinton Agar (Difco) plates, pH 7.2 and grown for an additional 24 h at 37 °C. Following incubation, the plates were examined for formation of *S. aureus* colonies. The concentration of ceftriaxon that produced no growth was recorded as the MBC.

*S. aureus* drug comparative efficacy measurements were obtained from the average MBC of each drug brand using ANOVA test and the statistical program SPSS 19. The relationship between efficacy and prices was determined by the Pearson’s correlation coefficient test and differences between price and efficacy of registered and non-registered brands compared by Student’s t-test. A *p* value ≤ 0.05 was considered statistically significant (drug brands: n = 40; sample replicates: 10 samples/drug brand; technical replicates: 10/samples; total MBC assays: 400).

## Results

**Fig. 1:**
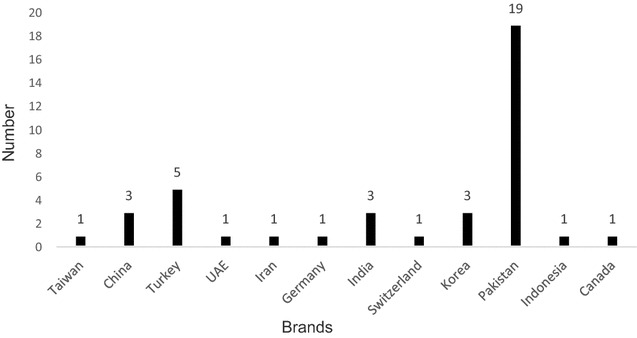
The* graph* shows number of brands of ceftriaxone tested and countries of manufacture. The *X*-axis indicates the various countries that the antibiotics were obtained from and the *Y*-axis, number of brands tested for ceftriaxone from those countries with the exact numbers given above each *bar*

In this study, a total of 40 different brands of ceftriaxone were obtained from Kabul’s main pharmacy to test price versus efficacy. Prices among the samples varied from 20 Afghanis (~0.35 USD) to 270 Afghanis (~4.69 USD) with an average price of 69.80 and a median of 57.50 Afghanis (STD ± 49.53). The ceftriaxone brands were manufactured in many countries, with most from Pakistan (19 companies), Turkey (5 companies), Korea (3 companies), China (3 companies), and India (3 companies). Others among the group represented by one brand each were manufactured in Canada, Germany, Taiwan, Indonesia, Iran, Switzerland and the United Arabic Emirates (Fig. 1).

We found that all of the ceftriaxone brands were packed according to international regulation standards and each vial contained distilled water for solubilizing the lyophilized drug. All vials were also labelled with the manufacturers’ name, batch number, date of manufacture, expiration date, and other specifications related to manufacturing.

**Fig. 2:**
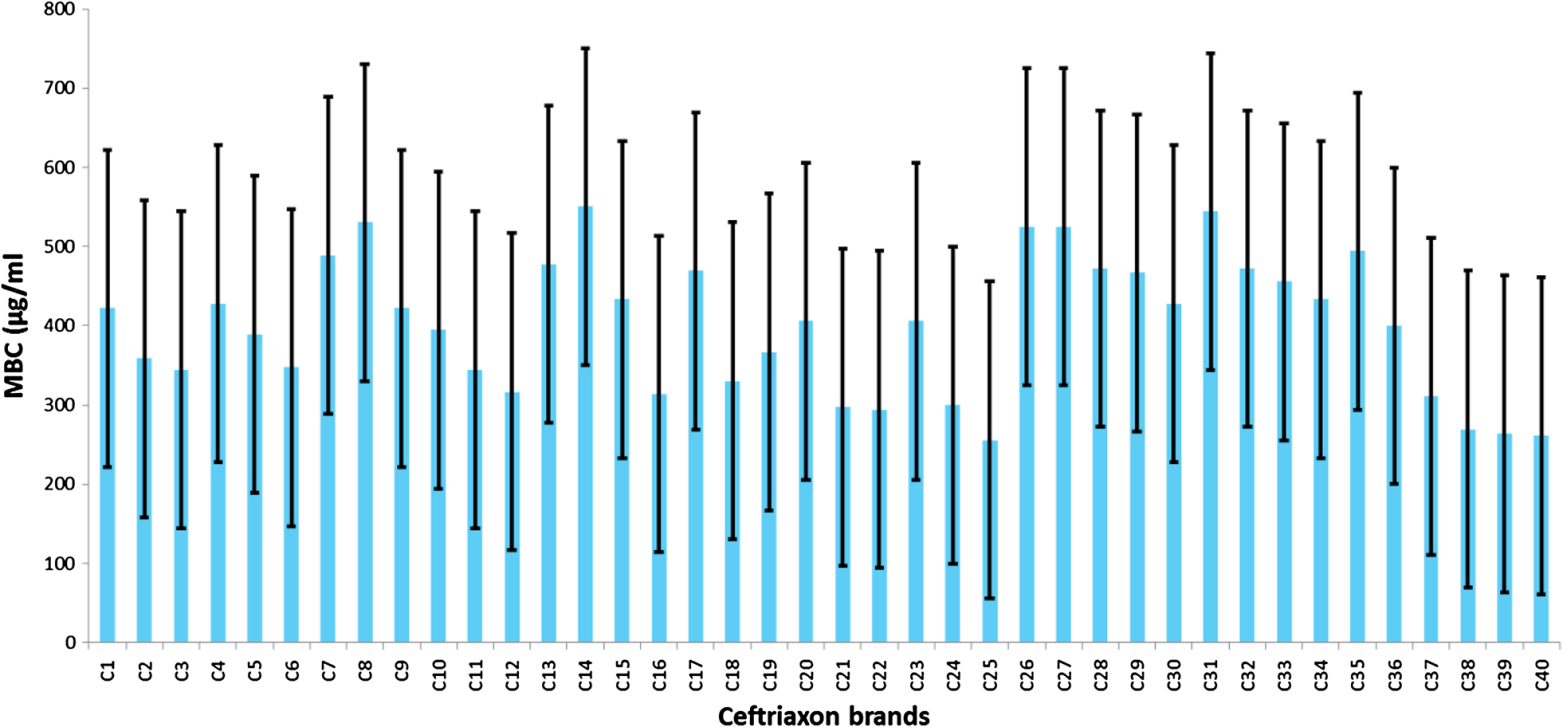
The* graph* shows the average minimal bactericidal concentration (MBC) of different brands of ceftriaxone against *S. aureus* in µg/ml. The *X*-axis indicates various brands of ceftriaxone and the *Y*-axis, the average of minimal bactericidal concentration/ml for each brand with minimum and maximum values indicated with *error bars*

Using ANOVA test we showed that the average Minimal Bactericidal Concentration for all brands against a sensitive strain of wild type *S. aureus* was 402.01 μg/ml (95 % CI, 373.13–430.90). The average MBC among brands ranged between 255.56 and 622.22 μg/ml but these differences were not statistically significant (*p* = 0.59) (Fig. 2).

**Fig. 3:**
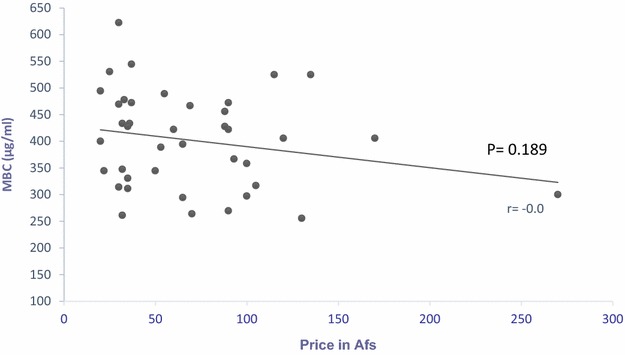
The* graph* shows correlation between price and MBC of each brands. The *X*-axis indicates price and the *Y*-axis the average minimal bactericidal concentration/per ml for each drug brand

Pearson test showed a poor correlation between price and efficacy among the various brands of ceftriaxone. A rise in the price by one Afs and a decrease in MBC of ~0.069 μg/ml was observed, but this was not statistically significant (r = −0.069, *p* = 0.189) (Fig. 3).

**Fig. 4:**
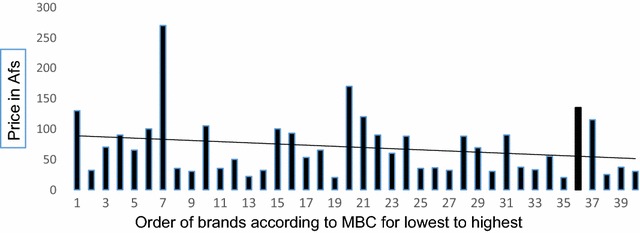
The* graph* shows the grading of different brands of ceftriaxone according to the MBC obtained versus price. The *X-*axis indicates the minimal bactericidal concentration of all brands from lowest to highest and the *Y*-axis, the price for each brand

Each brand of the ceftriaxone drug was also graded according to their respective MBC from lowest to highest values (1–40) versus price, however, our data again showed that there was no significant relationship between price and efficacy (Fig. 4).

Finally, we separated registered from unregistered brands and compared average prices and MBC using Student’s t test. We found that the average MBC for registered brands were 398.89 μg/ml while unregistered brands had an average MBC of 411.39 μg/ml (*p* = 0.71). The average price for ceftriaxone brands that were registered was 73.90 Afghanis versus 75.50 Afghanis for unregistered brands (*p* = 0.78). In both cases the results were not statistically significant.

## Discussion

Affordability of medical treatment is a key factor in patient health care globally and is especially true for those living in socioeconomically disadvantaged environments. The focus of this study was to test efficacy of several brands of Ceftriaxone sodium antibiotics as they relate to price using the MBC test for efficacy. We have not measured impurities in these samples and additional chemical tests will be required to do so. The results from our work show no significant differences in efficacy and prices of various ceftriaxone brands circulating in Kabul’s main Pharmacy chain. The findings are important to medical practice globally and indicate that clinicians should be prescribing lower cost ceftriaxone.

We also show that broth dilution antimicrobial susceptibility testing using the MBC method is an effective way to identify counterfeit drugs and substandard ones with diluted active ingredients [9]. According to the United States Pharmacopeia, the quantity of Ceftriaxone sodium antibiotics in labels must be in the range of 90–115 % [10]. All 40 brands of ceftriaxone used in this study were labelled with the active ingredient within this range and all showed the same efficacy against *S. aureus* regardless of price.

This study to show efficacy versus price is the first to be carried out in Afghanistan. Most of the brands included in our analyses were not studied before and none were tested for efficacy using the MBC assay. Our results however, validated other published work, which tested 3–6 of the brands used in our study by other methods, that the price of medically used drugs are not necessarily a determining factor for quality [11, 12].

One study conducted in Afghanistan of 11 different types of drugs available in public and private sector, including two brands of ceftriaxone found that 9 % of the drugs tested by chemical assays were substandard but ceftriaxone was not among the substandard group (13). This study concluded that the majority of drugs in Afghanistan are not counterfeit or substandard. The Pakistan cost-effective analysis study done with the six brands of ceftriaxone also showed similar in vitro activity of these brands against several gram negative and gram positive bacteria including *S. aureus* [11]. Assessment of 33 brands of ceftriaxone quality, available in Pakistan, by high-pressure liquid chromatography (HPLC) indicated that ~15.62 % ceftriaxone brands did not meet specification standards although none of the 96 samples of drugs tested were counterfeit [12]. In addition, studies in Nepal on the in vitro activity of three brands of ceftriaxone against various bacteria isolates from clinics in the country indicated that the minimal inhibitory concentration and biological activity among them were not the same and in one brand, MIC was higher by at least two fold suggesting this brand had less in vitro activity against isolates tested [14].

Our study which was done to test efficacy versus price of a much larger selection of ceftriaxone brands using biological methods argue against this finding and additionally showed that there was no difference in price versus efficacy among brands.

The results from our work may serve as a deterrent to both the manufacturer and import of substandard and counterfeit injectable antibiotics in Afghanistan, which will lower the risk of severe side effects caused by these drugs to patients.

We suggest that the differences in price for ceftriaxone are related to other factors that do not directly contribute to efficacy or quality of the antibiotic. Prices were found to be highest for drugs manufactured by large internationally known pharmaceutical companies, are in the medium-ranges when obtained from large local or regionally known generic pharmaceutical companies, and in the low-priced range from generic companies that sell drugs in regional markets only. Lack of monitoring and control of price rates of medical products in Afghanistan may play a role in the price range diversity observed in the country as importers and sellers purchase products with different prices. Thus, manufacturing cost, location of the company, as well as import and distribution costs may all contribute to fluctuations in price of the drugs rather than their potency. In addition, we cannot rule out the influence of patient preferences for specific brands and the general belief that higher prices means better quality as other contributing factors to price variations among these antibiotic brands.

## Conclusions

Our study shows that there is no relationship between cost and efficacy of various brands of ceftriaxone antibiotics in Kabul. While further studies using chemical assays for quality control tests including impurities and clinical trials are necessary to compare in vivo efficacy of the various ceftriaxone brands, based on our results, we recommend that physicians and pharmacists consider cheaper ceftriaxone alternatives for patients.
